# Denver and Marshall scores successfully predict susceptibility to multiple independent infections in trauma patients

**DOI:** 10.1371/journal.pone.0232175

**Published:** 2020-04-29

**Authors:** Marianna Almpani, Amy Tsurumi, Thomas Peponis, Yashoda V. Dhole, Laura F. Goodfield, Ronald G. Tompkins, Laurence G. Rahme

**Affiliations:** 1 Department of Surgery, Massachusetts General Hospital and Harvard Medical School, Boston, Massachusetts, United States of America; 2 Shriners Hospitals for Children-Boston, Boston, Massachusetts, United States of America; 3 Department of Microbiology and Immunobiology, Harvard Medical School, Boston, Massachusetts, United States of America; John Hunter Hospital and University of Newcastle, AUSTRALIA

## Abstract

Trauma patients are at risk of repeated hospital-acquired infections, however predictive scores aiming to identify susceptibility to such infections are lacking. The objective of this study was to investigate whether commonly employed disease-severity scores can successfully predict susceptibility to multiple independent infectious episodes (MIIEs) among trauma patients. A secondary analysis of data derived from the prospective, longitudinal study “Inflammation and the Host Response to Injury” (“Glue Grant”) was performed. 1,665 trauma patients, older than 16, were included. Patients who died within seven days from the time of injury were excluded. Five commonly used disease-severity scores [Denver, Marshall, Acute Physiology and Chronic Health Evaluation II (APACHE II), Injury Severity Score (ISS), and New Injury Severity Score (NISS)] were examined as independent predictors of susceptibility to MIIEs. The latter was defined as two or more independent infectious episodes during the index hospital stay. Multivariable logistic regression was used for the statistical analysis. 22.58% of the population was found to be susceptible to MIIEs. Denver and Marshall scores were highly predictive of the MIIE status. For every 1-unit increase in the Denver or the Marshall score, there was a respective 15% (Odds Ratio:1.15; 95% CI: 1.07–1.24; p < 0.001) or 16% (Odds Ratio:1.16; 95% CI: 1.09–1.24; p < 0.001) increase in the odds of MIIE occurrence. APACHE II, ISS, and NISS were not independent predictors of susceptibility to MIIEs. In conclusion, the Denver and Marshall scores can reliably predict which trauma patients are prone to MIIEs, prior to any clinical sign of infection. Early identification of these individuals would potentially allow the implementation of rapid, personalized, preventative measures, thus improving patient outcomes and reducing healthcare costs.

## Introduction

Traumatic injury has long been identified as one of the leading causes of morbidity and mortality worldwide [1]. The rates of hospital-acquired infections are high in the trauma population and such infections lead to a substantial increase in the hospital length of stay and associated costs [2–4].

Despite the plethora of available scoring systems that quantify the severity of trauma and organ failure and predict mortality following traumatic injury, only a limited number of studies have looked into the ability of these tools to calculate the risk of infections in trauma patients [5–8]. Frequently, these studies have concluded contradictory findings; some have shown that these scores can successfully predict the risk of hospital acquired infections [7, 8], whereas others have concluded a lack of predictive value in the trauma setting [5, 6]. Thus, the role of disease-severity scores in predicting infections following trauma is yet to be determined. More importantly, the existing studies do not take into account the fact that some patients might be susceptible to multiple infections, hence at risk of developing multiple independent infectious episodes (MIIEs) during their hospital stay. Patients that are prone to MIIEs represent a different cohort than those who develop either one or no infections. Identifying susceptibility to multiple infections could prove paramount and practically alter the management of such patients [9–11].

This study aims to evaluate whether organ dysfunction and trauma severity, assessed by commonly employed disease-severity scoring systems, could successfully predict patient susceptibility to MIIEs during their hospital stay. Early identification of trauma patients that are susceptible to MIIEs prior to any clinical evidence of infection, would allow for timely prophylactic interventions. Therefore, these scoring systems could prove to be valuable tools in our efforts to (i) prevent infections and the ensuing adverse and often life-threatening, complications; (ii) implement antibiotic stewardship in the era of rapidly emerging resistance to most current antimicrobial therapeutics; (iii) minimize the ever-rising health care costs.

## Methods

This study is a retrospective review of clinical data derived from the “Inflammation and the host response to injury” study (“Glue Grant”). Permission for this secondary use of the deidentified data was obtained from our Institutional Review Board (MGH IRB protocol 2008-P-000629/1).

“Inflammation and the host response to injury” is a multicenter, prospective, longitudinal study, that registered acutely injured patients, who were hospitalized at seven Level 1 trauma centers across the United States between 2003 and 2009. Among 1,937 patients in the Glue Grant trauma database with available relevant clinical data, 1,892 were adult (> 16 years) patients. From these, 227 patients died within 7 days from the date of injury and were excluded. Therefore, a population of 1,665 adult trauma patients was used for the analysis.

Eligible patients were stratified based on their susceptibility to multiple infections (suffered ≥ 2 independent infectious episodes), according to a previously developed decision tree [11]. Based on this previously published algorithm, the time of infection, the type of illness, and the microorganism causing the infection are the necessary elements to determine whether an infectious episode is independent of the other infections that a given patient has suffered, as described in [11]. Those with 2 or more independent infections were considered to be susceptible to MIIEs, and those who presented with either none or one infection were classified as non-susceptible to MIIEs. Each of the independent infectious episodes was classified as a surgical site infection (SSI), or as a non-SSI. Patients who experienced multiple infectious events presented with SSIs alone, non-SSIs alone, or a combination of both.

The definitions of the clinical outcomes of the patients were based on the guidelines outlined by the Glue Grant Consortium [12]. Accordingly, patients were deemed to have non-SSIs if they were diagnosed with pneumonia, ventilator-associated pneumonia, bloodstream infections, catheter-related bloodstream infections, urinary tract infections, meningitis, sinusitis, endocarditis, cholecystitis, empyema, and/or pseudomembranous colitis. SSIs included superficial incisional (referring to skin, or subcutaneous tissue infections), deep incisional (referring to infections of deep soft tissues, such as fascial and muscle layers), and organ/space SSIs [12].

Five commonly employed disease-severity scoring systems were captured in the Glue Grant trauma database and were compared between the case and the control groups. More specifically, we utilized (i) the Injury Severity Score (ISS), (ii) the New Injury Severity Score (NISS), (iii) the Acute Physiology and Chronic Health Evaluation II (APACHE II) score, (iv) the Denver score (S1 Table), and (v) the Marshall score (S2 Table; for the purposes of the Glue Grant, a modified Marshall score, excluding the neurologic component of the classic Marshall score, was utilized) [13, 14].

A univariate analysis was performed to identify possible risk factors that could contribute to the MIIE susceptibility status. We assessed all the available variables in the database including demographics [age, gender, race, body mass index (BMI)], comorbidities, tobacco smoking, alcohol and intravenous drug abuse, injury characteristics (type and mechanism), the elapsed time from the time of the injury to arrival in the Emergency Room (ER), the lowest systolic blood pressure (SBP) while the patient was in the ER, the hemoglobin level at the time of arrival in the ER, the ICU length of stay, and ICU interventions, if applicable (tracheostomy, length of mechanical ventilation). The Mann-Whitney U test was used to compare continuous variables, presented as the median ± interquartile range (25^th^ to 75^th^ percentile) and the chi-square was used for the comparison of categorical variables, presented as frequencies and percentages. A p-value of less than 0.05 was considered statistically significant.

Stepwise logistic regression analysis was subsequently performed. Variables that achieved a p-value of <0.2 were entered in the regression model. Multivariable analysis was performed with one scoring system at a time to determine the predictive value for each score. Adjusted odds ratios (aORs) and 95% confidence intervals (95% CI) were calculated. Statistical analysis was performed using the STATA software (version 13.1).

## Results

### Demographics and clinical characteristics of the population

The characteristics of the 1,665 adult trauma patients included in the study are displayed in Table 1. The median age of the cohort was 42.4 years, while the median BMI was 28.2. Approximately two thirds of the population were males (66.55%), and almost 9 out of 10 were of white race (89.37%). 28.95% were smokers, 13.27% admitted chronic alcohol abuse, while only 2.22% were intravenous drug users. Almost all patients had suffered blunt injuries alone (99.1%), with only a small number of subjects suffering a combination of both blunt and penetrating trauma (0.9%), while none exhibited penetrating injuries alone. The vast majority of the patients (84.74%) were involved in a Motor Vehicle Collision (MVC). The subjects were transferred to the hospital within a median of 1.9 hours. For those that fully recovered and were eventually discharged (1,582 patients), the median length of stay in the hospital was 21 days. 83 patients died. The latter had a median survival (hospital length of stay) of 9 days. All patients were admitted for at least one day in the ICU, with the median length of stay being 14.3 days.

**Table 1:** Characteristics of the population (n = 1,665) and comparison of patients who were susceptible to multiple infections to those who were not.

Variable		Data	Susceptibility Yes (n = 376)	Susceptibility No (n = 1289)	p-value
Age (in years), median (IQR)		42.4 (26–55)	41.64 (26–54)	42.69 (27–55)	0.373
BMI, median (IQR)		28.2 (23.88–31.25)	29.09 (24.21–32.42)	27.97 (23.79–30.93)	**0.008**
Gender, n (%)	Males	1,108 (66.55)	244 (64.89)	864 (67.03)	0.440
	Females	557 (33.45)	132 (35.11)	425 (32.97)	
Race, n (%)	White	1,488 (89.37)	333 (88.56)	1,155 (89.60)	0.707
	Asian	37 (2.22)	6 (1.60)	31 (2.40)	
	African American	101 (6.07)	27 (7.18)	74 (5.74)	
	Other	4 (0.24)	0 (0.00)	4 (0.31)	
	American Indian	21 (1.26)	6 (1.60)	15 (1.16)	
	Pacific Islanders	7 (0.42)	2 (0.53)	5 (0.39)	
	Unknown	7 (0.42)	2 (0.53)	5 (0.39)	
Hypertension, n (%)		259 (15.56)	60 (15.96)	199 (15.44)	0.807
Myocardial Infarction, n (%)		48 (2.88)	10 (2.66)	38 (2.95)	0.769
Congestive Heart Failure, n (%)		25 (1.5)	4 (1.06)	21 (1.63)	0.428
Atrial Tachyarrhythmias, n (%)		28 (1.68)	11 (2.93)	17 (1.32)	**0.033**
Ventricular Tachyarrhythmias, n (%)		4 (0.24)	0 (0.00)	4 (0.31)	0.279
Peripheral Vascular Disease, n (%)		17 (1.02)	6 (1.60)	11 (0.85)	0.208
Cerebrovascular Disease, n (%)		44 (2.64)	15 (3.99)	29 (2.25)	0.064
Dementia, n (%)		16 (0.96)	2 (0.53)	14 (1.09)	0.332
Seizure, n (%)		45 (2.70)	8 (2.13)	37 (2.87)	0.435
Previous Traumatic Brain Injury, n (%)		28 (1.68)	6 (1.60)	22 (1.71)	0.883
Parkinson’s Disease, n (%)		1 (0.06)	0 (0.00)	1 (0.08)	0.589
Chronic Pulmonary Disease, n (%)		54 (3.24)	14 (3.72)	40 (3.10)	0.550
Rheumatologic Disease, n (%)		24 (1.44)	3 (0.80)	21 (1.63)	0.234
Peptic Ulcer Disease, n (%)		21 (1.26)	6 (1.60)	15 (1.16)	0.509
Liver Disease, n (%)		57 (3.42)	14 (3.72)	43 (3.34)	0.716
Diabetes, n (%)		122 (7.33)	26 (6.91)	96 (7.45)	0.727
Hypothyroidism, n (%)		45 (2.70)	10 (2.66)	35 (2.72)	0.953
Hyperthyroidism, n (%)		4 (0.24)	0 (0.00)	4 (0.31)	0.279
Chronic Renal Dysfunction, n (%)		11 (0.66)	7 (1.86)	4 (0.31)	**0.001**
History of Malignancy, n (%)		54 (3.24)	13 (3.46)	41 (3.18)	0.790
Metastatic Solid Tumor, n (%)		4 (0.24)	2 (0.53)	2 (0.16)	0.189
Coagulopathy (congenital or acquired), n (%)		7 (0.42)	5 (1.33)	2 (0.16)	**0.002**
Smoker, n (%)		482 (28.95)	104 (27.66)	378 (29.33)	0.531
Chronic Alcoholism, n (%)		221 (13.27)	53 (14.10)	168 (13.03)	0.593
Intravenous Drug Use, n (%)		37 (2.22)	7 (1.86)	30 (2.33)	0.590
Psychiatric Disorder, n (%)		179 (10.75)	46 (12.23)	133 (10.32)	0.291
Recent Solid Organ Transplant, n (%)		5 (0.30)	2 (0.53)	3 (0.23)	0.351
Chemotherapy or Radiotherapy within 30 days, n (%)		2 (0.12)	0 (0.00)	2 (0.16)	0.445
Asthma, n (%)		86 (5.17)	22 (5.85)	64 (4.97)	0.495
Hypercholesterolemia / Hyperlipidemia, n (%)		98 (5.89)	22 (5.85)	76 (5.90)	0.974
Prior CABG, n (%)		58 (3.48)	15 (3.99)	43 (3.34)	0.543
Cardiovascular Disease, n (%)		16 (0.96)	2 (0.53)	14 (1.09)	0.332
Injury Type, n (%)	Blunt	1,650 (99.10)	373 (99.20)	1,277 (99.07)	0.810
	Blunt and penetrating	15 (0.90)	3 (0.80)	12 (0.93)	
Injury Mechanism, n (%)	Fall	142 (8.53)	33 (8.78)	109 (8.46)	0.839
	Machinery	36 (2.16)	7 (1.86)	29 (2.25)	
	MVC	1,411 (84.74)	323 (85.90)	1,088 (84.41)	
	Assault	44 (2.64)	8 (2.13)	36 (2.79)	
	Other	32 (1.92)	5 (1.33)	27 (2.09)	
Blood transfusion prior to blood sample collection, n (%)		438 (26.31)	94 (25.00)	344 (26.69)	0.513
ICU tracheostomy, n (%)		432 (25.95)	189 (50.27)	243 (18.85)	**<0.001**
ICU Days, median (IQR)		14.3 (5–19)	25.24 (16–31)	11.06 (4–15)	**<0.001**
ICU Ventilation Days, median (IQR)		10.5 (3–15)	19.77 (11–25)	7.77 (2–11)	**<0.001**
Time from injury to ER arrival (hours), median (IQR)		1.9 (0.8–2.7)	1.71 (0.7–2.1)	1.99 (0.8–2.8)	**0.001**
Lowest SBP at ER, median (IQR)		85.5 (72–97)	81.73 (69,5–94)	86.62 (74–98)	**<0.001**
Initial Hemoglobin at ER, median (IQR)		11.47 (9.8–13.2)	11.10 (9.3–13)	11.58 (10–13.2)	**0.001**
Major Procedure, n (%)		1,569 (94.23)	361 (96.01)	1,208 (93.72)	0.093
Max Marshal Score, median (IQR)		5.16 (3.29–6.64)	6.90 (5.21–8.28)	4.66 (2,981–6.01)	**<0.001**
Max Denver Score, median (IQR)		2.09 (0–3)	3.32 (2–5)	1.74 (0–3)	**<0.001**
APACHE II Score, median (IQR)		27.75 (24–33)	30.83 (27–35)	26.86 (23–32)	**<0.001**
ISS, median (IQR)		31.9 (22–41)	35.66 (27–43)	30.81 (22–41)	**<0.001**
NISS, median (IQR)		37.4 (27–48)	41.78 (34–50)	36.17 (27–45)	**<0.001**

n, number of observations; IQR, interquartile range (25^th^ to 75^th^ percentile); BMI, body mass index; CABG, coronary artery bypass graft; MVC, motor vehicle collision; ICU, intensive care unit; ER, emergency room; SBP, systolic blood pressure; APACHE II, acute physiology and chronic health evaluation II score; ISS, injury severity score; NISS, new injury severity score.

**Fig 1:**
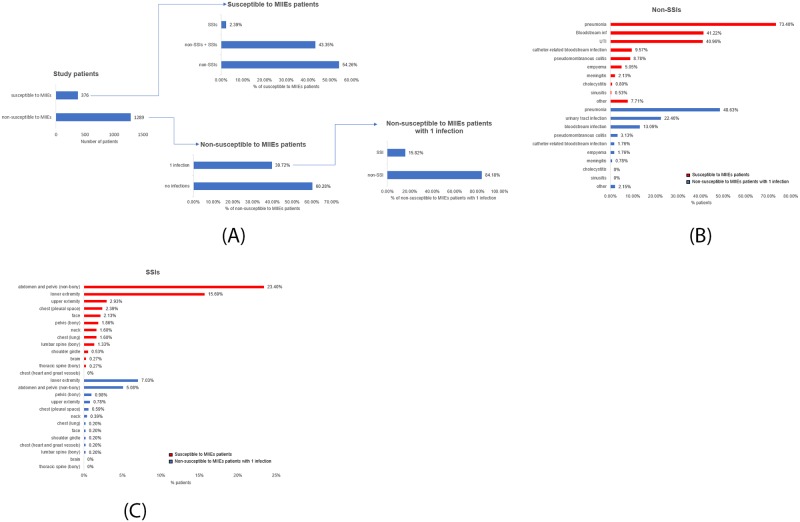
Characterization of the infections. **A**. Out of the 376 susceptible to MIIEs patients, 54.26% (n = 204) presented with non-surgical-site infections (non-SSIs) alone, 2.39% (n = 9) presented with surgical site infections (SSIs) alone, while 43.35% (n = 163) presented with both non-SSIs and SSIs. From the 1289 non-susceptible to MIIEs patients, 60.28% (n = 777) exhibited no infections and 39.72% (512) suffered 1 infection. Out of the 512 non-susceptible to MIIEs patients that presented with 1 infection, 84.18% (n = 431) suffered a non-surgical-site infection (non-SSI), while 15.82% (n = 81) suffered a surgical site infection (SSI). **B**. Characterization of the types of non-SSIs among susceptible to MIIEs patients (red bars) and non-susceptible to MIIEs patients with 1 infection (blue bars). **C**. Characterization of the location of SSIs among susceptible to MIIEs patients (red bars) and non-susceptible to MIIEs patients with 1 infection (blue bars).

Approximately one fifth of the subjects suffered two or more independent infectious episodes (22.58%) and were thus deemed to be susceptible to MIIEs. Out of the 376 susceptible to MIIEs patients, 54.26% presented with non-SSIs alone, 2.39% presented with SSIs alone, while 43.35% presented with both non-SSIs and SSIs (Fig 1A). From the non-susceptible to MIIEs patients, 60.28% exhibited no infections (Fig 1A), while among those with one infection, 84.18% presented with a non-SSI alone, and 15.82% presented with an SSI alone (Fig 1A). Pneumonia was the most common type of non-SSIs among both susceptible (73.40%) and non-susceptible to MIIEs patients (48.63%) (Fig 1B). Bloodstream and urinary infections followed in both groups, with 41.22% and 40.96% of the susceptible to MIIEs patients developing bloodstream and urinary infections respectively (Fig 1B), and 22.46% and 13.09% of the patients with 1 infection episode presenting with a urinary or bloodstream infection respectively (Fig 1B). The most usual locations for SSIs in both groups were the abdomen/pelvis area and the lower extremities, with abdomen/pelvis infections taking the lead in the susceptible to MIIEs group (23.40%) and lower extremity infections being the majority in the non-susceptible to MIIEs patients with 1 infection (7.03%) (Fig 1C).

The 3^rd^ and 4^rth^ columns of Table 1 present respectively the characteristics of the patients that were or were not susceptible to MIIEs. People who were susceptible to MIIEs tended to have a higher BMI, lower systolic blood pressure and hemoglobin levels in the ER, longer ICU stays, higher tracheostomy rates, prolonged need for mechanical ventilation, and higher rates of major procedures. Furthermore, in the univariate analysis, all the scoring systems examined were significantly higher for the subjects that suffered MIIEs, compared to those who suffered one or no infectious episodes (p<0.001 for all scoring systems).

### Evaluation of severity scores as independent predictors for MIIE susceptibility

The two multivariable analysis models including (a) the Denver score (Table 2), and (b) the Marshall score (Table 3), show that both scores are independent predictors of susceptibility to MIIEs (aOR = 1.15, p<0.001, 95% CI = 1.07–1.24 for the Denver score; aOR = 1.16, p<0.001, 95% CI = 1.09–1.24 for the Marshall score), while, in both models, ICU length of stay (aOR = 1.07, p<0.001, 95% CI = 1.03–1.10 in both models), and initial hemoglobin level upon arrival to the ER (aOR = 0.93, p = 0.010, 95% CI = 0.89–0.98 in the Denver model; aOR = 0.94, p = 0.015, 95% CI = 0.89–0.99 in the Marshall model) were also independent predictors of predisposition to MIIEs. Importantly, these results show that the odds of a patient to develop MIIEs increases by 15% with each unit increase in the Denver score and by 16% with each unit increase in the Marshall score.

**Table 2:** Multivariable analysis to find independent predictors of susceptibility to multiple infections (Denver score).

Variable	Odds Ratio	95% Confidence Interval	p-value
Denver score	1.15	1.07–1.24	<0.001
BMI	1.00	0.98–1.02	0.950
Atrial Tachyarrhythmias	1.34	0.51–3.57	0.551
Cerebrovascular Disease	1.28	0.59–2.78	0.533
Metastatic Solid Tumor	3.12	0.42–23.18	0.267
Chronic Renal Dysfunction	2.86	0.67–12.18	0.155
Coagulopathy congenital or acquired	4.79	0.66–34.54	0.120
ICU Days	1.07	1.03–1.10	<0.001
ICU Ventilation Days	1.02	0.99–1.06	0.191
ICU tracheostomy	1.25	0.90–1.75	0.187
Time from injury to ER arrival	0.92	0.84–1.01	0.072
Lowest SBP at the ER	1.00	0.99–1.01	0.876
Initial Hemoglobin value at the ER	0.93	0.89–0.98	0.010
Major Procedures	1.73	0.90–3.34	0.102

BMI, body mass index; ICU, intensive care unit; ER, emergency room; SBP, systolic blood pressure.

**Table 3:** Multivariable analysis to find independent predictors of susceptibility to multiple infections (Marshall).

Variable	Odds Ratio	95% Confidence Interval	p-value
Marshall score	1.16	1.09–1.24	<0.001
BMI	1.00	0.98–1.02	0.820
Atrial Tachyarrhythmias	1.33	0.51–3.46	0.561
Cerebrovascular Disease	1.28	0.59–2.78	0.533
Metastatic Solid Tumor	2.5	0.34–18.19	0.370
Chronic Renal Dysfunction	2.98	0.69–12.88	0.144
Coagulopathy congenital or acquired	4.76	0.66–34.51	0.123
ICU Days	1.07	1.03–1.10	<0.001
ICU Ventilation Days	1.02	0.98–1.06	0.305
ICU tracheostomy	1.30	0.93–1.81	0.129
Time from injury to ER arrival	0.92	0.84–1.00	0.060
Lowest SBP at the ER	1.00	0.99–1.01	0.855
Initial Hemoglobin value at the ER	0.94	0.89–0.99	0.015
Major Procedures	1.60	0.83–3.08	0.162

BMI, body mass index; ICU, intensive care unit; ER, emergency room; SBP, systolic blood pressure.

Variables that were removed from the stepwise logistic regression, because of p-values > 0.2 included: age, gender, race, hypertension, myocardial infarction, congestive heart failure, ventricular tachyarrhythmias, peripheral vascular disease, dementia, seizure, previous traumatic brain injury, Parkinson’s disease, chronic pulmonary disease, rheumatologic disease, peptic ulcer disease, liver disease, diabetes, hypothyroidism, hyperthyroidism, history of malignancy, smoking status, chronic alcoholism, intravenous drug use, psychiatric disorder, resent solid organ transplant, chemotherapy or radiotherapy within 30 days, asthma, hypercholesterolemia/hyperlipidemia, prior CABG, cardiovascular disease, injury type, injury mechanism, blood transfusion prior to blood sample collection.

Variables that were removed from the stepwise logistic regression, because of p-values > 0.2 included: age, gender, race, hypertension, myocardial infarction, congestive heart failure, ventricular tachyarrhythmias, peripheral vascular disease, dementia, seizure, previous traumatic brain injury, Parkinson’s disease, chronic pulmonary disease, rheumatologic disease, peptic ulcer disease, liver disease, diabetes, hypothyroidism, hyperthyroidism, history of malignancy, smoking status, chronic alcoholism, intravenous drug use, psychiatric disorder, resent solid organ transplant, chemotherapy or radiotherapy within 30 days, asthma, hypercholesterolemia/hyperlipidemia, prior CABG, cardiovascular disease, injury type, injury mechanism, blood transfusion prior to blood sample collection.

On the contrary, APACHE II, ISS, and NISS in their respective multivariable analysis models (S3–S5 Tables) failed to independently predict which patients would be more prone to MIIEs (aOR = 1.02, p = 0.097, 95% CI = 1.00–1.04 for APACHE II; aOR = 1.01, p = 0.321, 95% CI = 0.99–1.02 for ISS; aOR = 1.01, p = 0.131, 95% CI = 1.00–1.02 for NISS), with possible confounding factors in all three models being: a) the ICU length of stay (aOR = 1.07, p<0.001, 95% CI = 1.03–1.10 in the APACHE II model; aOR = 1.06, p<0.001, 95% CI = 1.03–1.10 in the ISS and the NISS models), b) the ICU ventilation duration (aOR = 1.04, p = 0.043, 95% CI = 1.00–1.07 in the APACHE II model; aOR = 1.04, p = 0.026, 95% CI = 1.00–1.08 in the ISS model; aOR = 1.04, p = 0.031, 95% CI = 1.00–1.08 in the NISS model), and the initial hemoglobin level upon arrival to the ER (aOR = 0.94, p = 0.021, 95% CI = 0.89–0.99 in the APACHE II model; aOR = 0.93, p = 0.008, 95% CI = 0.88–0.98 in the ISS and the NISS models).

### Validation of the Denver and Marshall scores’ ability to predict susceptibility to MIIEs among patients with early-assigned scores

While the ISS and NISS scores were assigned to all patients upon hospital admission, and the APACHE II score was assigned within 24h from admission to the Intensive Care Unit (ICU), the Denver and Marshall scores were assigned at different time points for different patients during their recovery course. To control for the time that the Denver and Marshall scores were assigned, subsequent stepwise logistic regression was performed, including only those patients that were assigned a Denver or a Marshall score within 3 days from admission to the hospital. This time point was deemed to be early, as it preceded the average day of infection occurrence, with non-SSIs occurring on average around day 10 and SSIs occurring on average around day 13 of hospitalization. For the validation of our Denver score results, 1282 patients that had an early-assigned Denver score were included, 225 of which were susceptible to MIIEs and 1057 were not. For the validation of our Marshall score results, 1109 patients that had an early-assigned Marshall score were included, 174 of which were susceptible to MIIEs and 935 were not.

The two multivariable analysis models including (a) the Denver score (Table 4), and (b) the Marshall score (Table 5), show that both scores, even when assigned early, are independent predictors of susceptibility to MIIEs (aOR = 1.16, p = 0.014, 95% CI = 1.03–1.31 for the Denver score; aOR = 1.30, p<0.001, 95% CI = 1.15–1.46 for the Marshall score). ICU length of stay continued to be an independent predictor of predisposition to MIIEs in both models (aOR = 1.11, p<0.001, 95% CI = 1.06–1.16 in the Denver model); aOR = 1.09, p<0.001, 95% CI = 1.04–1.15 in the Marshall model). Initial hemoglobin level upon arrival to the ER remained an independent predictor only in the Denver model (aOR = 0.92, p = 0.021, 95% CI = 0.86–0.99).

**Table 4:** Multivariable analysis to find independent predictors of susceptibility to multiple infections (early-assigned Denver).

Variable	Odds Ratio	95% Confidence Interval	p-value
Denver score	1.16	1.03–1.31	0.014
BMI	1.00	0.97–1.03	0.999
Atrial Tachyarrhythmias	2.06	0.63–6.73	0.231
Cerebrovascular Disease	1.77	0.64–4.86	0.271
Chronic Renal Dysfunction	4.09	0.73–22.97	0.110
Coagulopathy congenital or acquired	1.45	0.13–16.35	0.762
ICU Days	1.11	1.06–1.16	<0.001
ICU Ventilation Days	1.01	0.97–1.06	0.617
ICU tracheostomy	1.24	0.79–1.95	0.351
Time from injury to ER arrival	0.99	0.88–1.11	0.809
Lowest SBP at the ER	1.00	0.99–1.01	0.606
Initial Hemoglobin value at the ER	0.92	0.86–0.99	0.021
Major Procedures	1.74	0.74–4.07	0.201

**Table 5:** Multivariable analysis to find independent predictors of susceptibility to multiple infections (early-assigned Marshall).

Variable	Odds Ratio	95% Confidence Interval	p-value
Marshall score	1.30	1.15–1.46	<0.001
BMI	0.99	0.96–1.02	0.424
Atrial Tachyarrhythmias	1.42	0.33–6.10	0.640
Cerebrovascular Disease	1.29	0.38–4.41	0.682
Chronic Renal Dysfunction	4.25	0.46–39.49	0.203
Coagulopathy congenital or acquired	4.53	0.08–272.30	0.470
ICU Days	1.09	1.04–1.15	<0.001
ICU Ventilation Days	1.02	0.96–1.07	0.579
ICU tracheostomy	1.37	0.81–2.31	0.241
Time from injury to ER arrival	0.92	0.81–1.06	0.246
Lowest SBP at the ER	1.00	1.00–1.01	0.337
Initial Hemoglobin value at the ER	0.92	0.85–0.99	0.847
Major Procedures	1.98	0.64–6.07	0.645

## Discussion

Our study shows that the Denver and Marshall scores, which are used to assess the severity of organ failure in the setting of traumatic injury, can accurately predict susceptibility to MIIEs after trauma, even when assigned much earlier than infection occurrence. These findings suggest that Denver and Marshall could potentially prove to be invaluable tools in predicting the predisposition of trauma patients to MIIEs, before the latter develop any clinical signs of infection, therefore altering the clinical management and leading to implementation of prophylactic measures. Such a predictive tool is currently lacking.

Previous studies investigating the potential of trauma assessment scoring systems to identify patients at increased risk of post-traumatic hospital-acquired infections mainly focused on scores such as APACHE II, ISS and NISS. Hurr and colleagues [6], reported that APACHE II and ISS did not successfully predict the incidence of infections in trauma patients. All the same, Cheadle and colleagues [5] reported that ISS failed to distinguish between trauma patients with and without infections. These results are in accordance with our findings. Those studies were somewhat limited by their relatively low sample size, as well as by their monocentric nature. Our multicentric study, with more than 1,660 trauma patients, confers sufficient sample size, decreasing the risk of a Type 2 error, thus providing more confidence regarding the aforementioned observations. Furthermore, pediatric patients were included in Hurr’s study [6], despite the fact that the anatomic aspects of pediatric trauma and the physiologic responses to injury in this population significantly differ from those of their adult counterparts [15]. Two more recent prospective studies reported that ISS, NISS and APACHE II directly correlate to the risk of infections in trauma patients, findings that are not consistent with our results [7, 8]. However, in these studies several possible confounders, including those identified in our study, were not accounted for, hence causing a potentially spurious association between APACHE II / ISS / NISS and the presence of infections. Finally, to our best knowledge, there is no study investigating the ability of the Marshall and Denver scores, to predict infections in the afore-mentioned population.

Our finding that physiology-based disease-severity scores (i.e., Denver and Marshall), rather than anatomy-based trauma-severity scores (i.e., ISS and NISS) can successfully predict the increased post-traumatic predisposition to MIIEs, conceivably suggests that the post-traumatic susceptibility to MIIEs correlates better with the individual physiologic response to injury, rather than the anatomical severity of the trauma. A seemingly contradictory finding is the fact that even though APACHE II also utilizes physiologic aspects to assess disease severity, this score fails to identify the patients, who are at a higher risk of MIIEs. This observation could potentially be explained by the fact that APACHE II does not account for the hepatic function, aspect that is integrated in both the Denver and the Marshall scores, thus possibly implying a key role for liver dysfunction in the setting of post-injury infections. In support of this consideration, a recent publication describing 4 new sepsis phenotypes, suggests that the “δ phenotype” refers to a subgroup of patients, the members of which are more likely to have hepatic dysfunction, exert significant derangement of their immune responses and are more prone to adverse clinical outcomes [16].

As expected, blood loss after trauma seems to predispose patients to infections, as indicated by the finding that higher hemoglobin levels at the time of admission conferred protection in terms of susceptibility to MIIEs (aOR<1 for both Denver and Marshall: aOR = 0.93, p = 0.010, 95% CI = 0.89–0.98 for Denver; aOR = 0.94, p = 0.015, 95% CI = 0.89–0.99 for Marshall). Regional hypoxia and the subsequent immediate and persistent depression of various aspects of the immune response has been identified as the underlying mechanism of increased susceptibility to infections in the setting of acute hemorrhage [17]. Similarly, ICU length of stay was another independent predictor of MIIE susceptibility. This result was also anticipated, as it has long been clearly documented that longer duration of ICU stay leads to increased rates of infections [18]. The relationship between MIIEs and length of ICU stays, tracheostomy rates, duration of mechanical ventilation and number of major procedures would arguably be expected to be bidirectional. Therefore, prospective studies would confer higher levels of confidence in delineating this effect.

Our study has a number of strengths. First, an obvious advantage is that our findings derive from the analysis of prospectively collected data of more than 1,660 trauma patients from 7 institutions across the US. This confers sufficient variability and confidence to elucidate significant differences, while effectively identifying erroneous correlations. Second, the longitudinal nature of the data collection for the purposes of the “Glue Grant”, gave us the opportunity to develop an algorithm that effectively identifies patients who are susceptible to multiple infections, which clinically manifests as multiple independent infectious episodes [10, 11]. Though previous studies have interrogated the ability of disease-severity scores to predict the risk of infection, adoption of this decision tree for the first time in the trauma population gives us the opportunity to take this consideration one step further and assess the ability of these scores to identify patients at risk of numerous independent infections, a distinct outcome, with deleterious consequences for the patients and increased cost rates for the hospitals. Third, to our knowledge, no previous study has assessed the Denver and Marshall scores in the setting of post-traumatic infection prognostication, which according to our results, are the only scores that can reliably predict the MIIE occurrence.

Our study carries a few limitations. First, its retrospective nature may raise potential for selection bias. Second, our data derives only from Level 1 trauma centers, thus it may not be generalizable to the patient population of other institutions. Furthermore, given that the anatomical scores (ISS and NISS) are calculated based on the Abbreviated Injury Scale (AIS) that classifies the injuries by body region on a 6-point scale, it is possible that specific components might be more important than others. For instance, a traumatic brain injury (TBI) could arguably predispose to more infections compared to a leg injury. However, given that the ISS and NISS are already computed in the Glue Grant database and the individual components are not available, it was impossible to assess the impact of these individual body-part injury scores. Lastly, even though we controlled for a plethora of possible confounding factors that were available in the Glue Grant trauma database, it is possible that there are more lurking variables that were not captured, such as the type and complexity of any operations performed, that could possibly influence the results.

Taken together, our findings could potentially facilitate clinical decision-making by identifying patient subpopulations that are at higher risk of suffering multiple infectious episodes during their hospital stay. Early prognosis, before infections occur, could strategically guide the initiation timing and the duration of antibiotic administration to these patients. It would also allow physicians to implement prophylactic measures, enhance patient nutrition and formulate potent personalized treatment plans for this group of patients, thus protecting those at higher risk of repeated infections during their recovery period. Specifically, patients that are deemed to be susceptible to MIIEs could benefit from the use of central and peripheral IV lines, as well as urine catheters that are coated or impregnated with antimicrobial or antibiotic agents. Studies have shown that their use has been linked to a significant reduction in infection rates [19, 20]. Use of such devices in the wide trauma population would be unnecessarily expensive. However, implementation of this measure in the susceptible to MIIEs population will potentially be cost-effective. Furthermore, strict early replacement of IV lines and urine catheters in this population could be crucial in infection prevention. The time that such devices are removed or replaced differs widely, as replacing them when clinically indicated is more cost-effective than routine replacements every 72-94h [21]. However, the susceptible to MIIEs population could potentially benefit from early replacement, which would subsequently lower their infection rates as well as the related health-care costs. Moreover, implementation of immune-modulatory nutrition has been shown to be beneficial for critically ill patients, therefore it could potentially have a favorable effect in the susceptible to MIIEs population [22]. Finally, given the susceptibility of these patients to MIIEs, taking transmission-based precautions (contact, droplet and airborne) that are not used in the wide trauma population, could aid towards limiting the exposure of these patients to infectious agents. Such an approach would (i) reduce the transmission of multi-drug resistant microbes, (ii) promote antibiotic stewardship, (iii) prevent hospital-acquired infections with the subsequently high mortality and morbidity rates, (iv) shorten hospital length of stay, and (v) drastically reduce the cost of care. Further research combining these clinical-characteristic-based prediction models with gene expression profiling from blood samples of trauma patients could potentially confer even higher levels of confidence in our effort to predict which patients are susceptible to multiple infections. This combination could offer a precision medicine approach that would have the potential not only to limit infections before their onset, but also to reveal novel host immunomodulatory targets for infection treatment and prevention following severe trauma.

## Conclusions

Marshall and Denver scores can reliably predict the susceptibility of trauma patients to MIIEs during their hospital stay. Utilization of these scores could effectively identify patients that would benefit from prophylactic measures to avoid multiple infections and could therefore decrease the high rates of infection-related morbidity and mortality in the trauma population.

## Supporting information

S1 Table Denver score components. (DOCX)

S2 Table Marshall score components. (DOCX)

S3 Table Multivariable analysis to find independent predictors of hypersusceptibility to infections (APACHE II). (DOCX)

S4 Table Multivariable analysis to find independent predictors of hypersusceptibility to infections (ISS). (DOCX)

S5 Table Multivariable analysis to find independent predictors of hypersusceptibility to infections (NISS). (DOCX)
