# Supplementary material for: Denver and Marshall scores successfully predict susceptibility to multiple independent infections in trauma patients
Source: PLoS One. 2020 Apr 29;15(4):e0232175. doi: 10.1371/journal.pone.0232175 (PMC7190145; doi:10.1371/journal.pone.0232175)
Supplement: S2 Table — (DOCX) [file pone.0232175.s002.docx]

**S2 Table. Marshall score components**

| **Component** | **Measurement** |
| --- | --- |
| Respiratory | PaO2/FiO2 |
| Renal | Creatinine |
| Hepatic | Bilirubin |
| Cardiovascular | PAR^a^ |

^a^PAR, Pressure Adjusted Heart Rate.

Adopted from [14].
