# Supplementary material for: Denver and Marshall scores successfully predict susceptibility to multiple independent infections in trauma patients
Source: PLoS One. 2020 Apr 29;15(4):e0232175. doi: 10.1371/journal.pone.0232175 (PMC7190145; doi:10.1371/journal.pone.0232175)
Supplement: S3 Table — (DOCX) [file pone.0232175.s003.docx]

**S3 Table.** **Multivariable analysis to find independent predictors of hypersusceptibility to infections (APACHE II).**

| **Variable** | **Odds Ratio** | **95% Confidence Interval** | **p-value** |
| --- | --- | --- | --- |
| APACHE II | 1.02 | 1.00 – 1.04 | 0.097 |
| BMI | 1.00 | 0.98 – 1.02 | 0.789 |
| Atrial Tachyarrhythmias | 1.23 | 0.46 – 3.28 | 0.675 |
| Cerebrovascular Disease | 1.22 | 0.56 – 2.67 | 0.617 |
| Metastatic Solid Tumor | 2.89 | 0.38 – 21.98 | 0.305 |
| Chronic Renal Dysfunction | 3.15 | 0.75 – 13.30 | 0.118 |
| Coagulopathy congenital or acquired | 4.53 | 0.65 – 31.39 | 0.126 |
| ICU Days | 1.07 | 1.03 – 1.10 | <0.001 |
| ICU Ventilation Days | 1.04 | 1.00 - 1.07 | 0.043 |
| ICU tracheostomy | 1.17 | 0.84 – 1.64 | 0.343 |
| Time from injury to ER arrival | 0.92 | 0.84 – 1.00 | 0.057 |
| Lowest SBP at the ER | 1.00 | 0.99 – 1.01 | 0.861 |
| Initial Hemoglobin value at the ER | 0.94 | 0.89 – 0.99 | 0.021 |
| Major Procedures | 1.77 | 0.92 – 3.41 | 0.090 |
