# Supplementary material for: Denver and Marshall scores successfully predict susceptibility to multiple independent infections in trauma patients
Source: PLoS One. 2020 Apr 29;15(4):e0232175. doi: 10.1371/journal.pone.0232175 (PMC7190145; doi:10.1371/journal.pone.0232175)
Supplement: S4 Table — (DOCX) [file pone.0232175.s004.docx]

**S4 Table.** **Multivariable analysis to find independent predictors of hypersusceptibility to infections (ISS).**

| **Variable** | **Odds Ratio** | **95% Confidence Interval** | **p-value** |
| --- | --- | --- | --- |
| ISS | 1.01 | 0.99 – 1.02 | 0.321 |
| BMI | 1.00 | 0.98 – 1.03 | 0.686 |
| Atrial Tachyarrhythmias | 1.29 | 0.49 – 3.44 | 0.607 |
| Cerebrovascular Disease | 1.27 | 0.58 – 2.77 | 0.550 |
| Metastatic Solid Tumor | 3.39 | 0.45 – 25.71 | 0.237 |
| Chronic Renal Dysfunction | 3.63 | 0.86 – 15.28 | 0.079 |
| Coagulopathy congenital or acquired | 4.68 | 0.69 – 31.57 | 0.113 |
| ICU Days | 1.06 | 1.03 – 1.10 | <0.001 |
| ICU Ventilation Days | 1.04 | 1.00 - 1.08 | 0.026 |
| ICU tracheostomy | 1.17 | 0.84 – 1.63 | 0.354 |
| Time from injury to ER arrival | 0.92 | 0.84 – 1.00 | 0.055 |
| Lowest SBP at the ER | 1.00 | 0.99 – 1.01 | 0.990 |
| Initial Hemoglobin value at the ER | 0.93 | 0.88 – 0.98 | 0.008 |
| Major Procedures | 1.70 | 0.88 – 3.28 | 0.113 |
