# Supplementary material for: Denver and Marshall scores successfully predict susceptibility to multiple independent infections in trauma patients
Source: PLoS One. 2020 Apr 29;15(4):e0232175. doi: 10.1371/journal.pone.0232175 (PMC7190145; doi:10.1371/journal.pone.0232175)
Supplement: S5 Table — (DOCX) [file pone.0232175.s005.docx]

**S5 Table.** **Multivariable analysis to find independent predictors of hypersusceptibility to infections (NISS).**

| **Variable** | **Odds Ratio** | **95% Confidence Interval** | **p-value** |
| --- | --- | --- | --- |
| NISS | 1.01 | 1.00 – 1.02 | 0.131 |
| BMI | 1.00 | 0.98 – 1.03 | 0.656 |
| Atrial Tachyarrhythmias | 1.32 | 0.50 – 3.51 | 0.578 |
| Cerebrovascular Disease | 1.29 | 0.59 – 2.81 | 0.524 |
| Metastatic Solid Tumor | 3.39 | 0.45 – 25.73 | 0.237 |
| Chronic Renal Dysfunction | 3.69 | 0.88 – 15.43 | 0.074 |
| Coagulopathy congenital or acquired | 4.67 | 0.70 – 31.25 | 0.112 |
| ICU Days | 1.06 | 1.03 – 1.10 | <0.001 |
| ICU Ventilation Days | 1.04 | 1.00 - 1.08 | 0.031 |
| ICU tracheostomy | 1.16 | 0.83 – 1.62 | 0.371 |
| Time from injury to ER arrival | 0.92 | 0.84 – 1.00 | 0.056 |
| Lowest SBP at the ER | 1.00 | 0.99 – 1.01 | 0.991 |
| Initial Hemoglobin value at the ER | 0.93 | 0.88 – 0.98 | 0.008 |
| Major Procedures | 1.69 | 0.87 – 3.25 | 0.119 |
